# Towards Sustainable Use of Potassium in Pineapple Waste

**DOI:** 10.1100/tsw.2004.199

**Published:** 2004-11-20

**Authors:** Osumanu H. Ahmed, M.H.A. Husni, A.R. Anuar, M.M. Hanafi

**Affiliations:** Department of Land Management, Faculty of Agriculture, Universiti Putra Malaysia 43400 UPM Serdang, Selangor, Malaysia

**Keywords:** pineapple waste, agricultural products, K-humate, K-fulvate, pineapple leaves

## Abstract

Due to the 1997/98 haze problem in South-East Asia and the increasing need for sustainable food production and development, the usual management of crop residues (including pineapple wastes) through burning is prohibited. As a result, the need for alternative uses of pineapple wastes in pineapple production has been emphasized. This study investigated an environmentally friendly means of recycling pineapple leaves for agricultural use. Pineapple leaves were shredded and composted in a composting drum for 30 days. Part of the shredded leaves was ashed in a muffle furnace for 4 h. Humic acid (HA), K-fulvate, and K in HA and compost were analyzed using standard procedures. An ash to water ratio of 1:7 was used to extract 0.1 molar (M) KOH from the shredded leaves. The 0.1 M KOH contained 50% K and was able to extract 20% HA from the composted pineapple leaves. Percent K in the fulvate using 0.1 M KOH was 43. Besides serving as a foliar spray (supplement soil application K fertilizers), source of K for freshwater fish (e.g., tilapia), the HA produced can be used as a soil conditioner. Studies show that between 0.05—0–01 g of HA per kg soil retards runoff by 36% in sandy and sandy loam soils. The K-fulvate can be used as a fluid fertilizer. In addition, the pH of 2 of the K-fulvate suggests it could be used to dissolve phosphate rocks, particularly those in the arid regions where high soil pH does not facilitate the dissolution of these important rocks that serve as one of the sources of phosphorus fertilizer in agriculture.

## INTRODUCTION

In Malaysia, pineapples are largely cultivated on peat. Due to the low, load-bearing ability to support heavy-duty tillage implements, pineapple residues on peat are recycled through burning. Due to the ever-increasing awareness about environmental pollution and the need for sustainable agriculture, concerns have been expressed about the long-term effect of burning crop residues, of which pineapple residue burning is no exception. Burning does not only cause haze, but also causes peat burn that is difficult to control (perhaps one of the reasons why pineapple residue burning on peat has attracted more attention than rice straw burning on mineral soils). Alternatives to burning are *in situ* decomposition of pineapple residues or their removal before planting, but while the former leads to disease outbreaks, the latter has the problem of residue disposal.

Following the 1997 economic loss in the agricultural sector due to haze in South-East Asia[1,2,3], a comparison of the economic viability of burning pineapple residues and removal of these residues before replanting was carried out[4]. Burning of 4.34 Mg $ha^{-1}$ pineapple leaves per cropping season produces 1.31 Mg $ha^{-1}$ ash. The total amount of K in this ash is 240.43 kg K $ha^{-1}$. This high K does not increase K uptake and yield because the K easily gets leached[5]. Besides, Husni et al. observed[4] that the practice of removing pineapple residues before replanting was economically viable compared to burning the residues as the cost of air pollution associated with burning was estimated at US\$ 620 $ha^{-1}$ against the cost of US\$ 23.50 $ha^{-1}$ associated with residue removal.

This study, however, failed to address the issue of whom or how to manage the residues after removal. This pressing issue prompted a study to investigate the feasibility of leaving pineapple residues to decompose *in situ* rather than removing these residues only for them to become more of a liability than an asset. The study concluded that the *in situ* decomposition practice was economically viable[6]. But the problem with this practice is that about 5.5 Mg $ha^{-1}$ pineapple residue is produced per a cropping season (2 years) and if this large amount of residue is left to decompose *in situ*, it is anticipated that a prolonged adoption of this practice without a very careful handling of these residues may lead to fire, pests, and disease outbreaks. In the course of the study, it was observed that it took more than 10 months for the pineapple residues to start decomposing. It is therefore obvious that the possibility of the problem of adding more organic matter to a poorly decomposed peat with time is inevitable. In order that pineapple residues become more of an asset than a liability to farmers, this study investigated the possible way of developing K-rich products from pineapple leaves for agricultural use. This approach has the potential of being cost effective because, besides the fact that farmers are willing to dispose of their residues freely (a form of relief), the K-humate and K-fulvate can serve not only as a source, but the humic and fulvic acids have the ability to improve soil physical, chemical, and biological properties as well as forming complex with Al[7], a mechanism useful to reduce Al-toxicity-acid soils of Malaysia.

## MATERIALS AND METHODS

Pineapple leaves were collected from Simpang Rengam Pineapple Estate, Peninsula Pineapple Plantation, Simpang Rengam, Johor in the southern part of Malaysia. The leaves were air dried, shredded, and chipped using Briggs and Stratton shredder-chipper. Part of the shredded leaves was incinerated using standard procedures and the KOH of the ashed leaves extracted by dissolving it in distilled water for 24 h at a ratio of 1:7 (ash/water, weight/volume) that enabled the attainment of 0.10 *M* KOH (acid-base titration was used to confirm this concentration) solution suitable for the extraction of humic substances[8]. This 0.10 *M* KOH was directly analyzed for K, Ca, Mg, Na, and pH using atomic absorption spectroscopy and pH meter, respectively.

A 25-kg amount of shredded leaves was mixed with 1.25 kg chicken dung in liquid form (as source of microorganisms and nitrogen), and 1.25 kg chicken feed. The mixture was composted in a composting drum for 30 days, a period when the compost temperature equaled the ambient temperature (29˚C). The moisture content of the compost was maintained at 55–60%.

Standard procedures[9] were used to extract and purify humic acid (HA) (in compost) with some modifications. Five grams (dry-weight basis) of compost (at natural moisture level) were placed into a polyethylene centrifuge bottle and a 50-mL 0.10 *M* KOH[7] solution was added, and the bottle stoppered tightly with a rubber stopper. The mixture was equilibrated for 20 h at room temperature on a reciprocal mechanical shaker. The sides of the bottle were washed with distilled water and the mixture centrifuged at 10,000 rpm for 20 min. The dark-colored supernatant liquor was decanted and the pH of the solution adjusted to 1.0 with 6 *N* HCl.

The procedure was repeated with the addition of 50 mL distilled water to the compost and equilibrated for 1 h. The residue was dispersed in 50 mL of distilled water, centrifuged, and the supernatant liquor added to the previous extracts. The pH of the resulting solution was adjusted to 1 with 6 *N* HCl and the HA allowed to stand at room temperature for 24 h. The excess supernatant liquor (fulvic acid, FA)[10] was siphoned off from the acidified extract. The remainder of the suspension was transferred to a polyethylene bottle and the HA centrifuged off. The HA was then washed against distilled water and dried at 40$^{o}$C. The FA was directly analyzed for K, Ca, Mg, Na, and pH using the methods previously described. Functional-group (carboxylic and phenolic) analyses were conducted by the method described by Inbar et al.[11]. The entire experiment was replicated three times.

## RESULTS AND DISCUSSION

The pH, K, Ca, Mg, and Na of the 0.10 *M* KOH solution were approximately 11, 50, 0.03, 0.01, and 0.13%, respectively. The high K concentration in the 0.10 *M* KOH solution can be attributed to the high requirement of pineapple for K[12] and high K content in pineapple leaves[5]. Hence, by ashing these leaves, a unit weight of this ash will be high in K. The high uptake of K and other elements could also be that the roots of pineapple in peat were not very restricted or confined (as pineapple roots are sensitive soil compaction) to the tilled area because of the low bulk density of peat which allows the roots to access larger volume of water and plant nutrients. Under conducive situations, pineapple roots could spread up to 1–2 m laterally and 0.85 m in depth[13]. For economic reasons, the KOH was not further purified because this will involve some cost and hence rendering this method of KOH production expensive. Besides, elements such as K, Ca, Mg, and Na in the KOH could be recycled as fulvate.

The purity of HA is essential in the chemical characterization. The effect of the washing of HA on K, Ca, Mg, and Na are shown in Figs. 1, 2, 3, and 4. Washing generally reduced the contents of these elements. However, where the primary aim is to recycle HA or the salts of HA, it looks better not to wash the HA. This is because, in this study, the unwashed HA (humate or salt of HA) contains some essential elements (particularly K) that could serve as plant nutrient supplement when KOH is derived from pineapple leaves. In this study, the unwashed HA extracted contained 6% K and the pattern of its K release in distilled water with time is shown in Fig. 5. About 4.50% K was water soluble within a day and leveled off at about 4% and this observation remained relatively constant throughout the period of the study; implying that not more than between 4.5–4% K can be released if the unwashed HA is used.

This characteristic may be used in freshwater fish production, as the soluble K in the K-humate exceeds the freshwater fish requirement. Fresh water contains less than 10 mg K $L^{-1}$[14]. According to Shearer[15], inadequate levels of a dietary K supply for young king salmon (freshwater fish) resulted in poor feed intake and feed utilization as well as growth retardation when the diet contained less than 1 mg K $L^{-1}$, and increased mortality also occurred. Maximum growth was obtained at a dietary level of 8 g K $kg^{-1}$ dry diet, while whole-body concentrations were normal at 6–12 g K $kg^{-1}$ diet[15]. Besides, the backbone of K-humate, which is HA, can stimulate the growth of organisms, such as phytoplanktons and algae[16], diatoms, and dinoflagellates. These organisms play an important role in the food chain of fish. It is believed that HA stimulate the growth of these organisms by chelating and solubilizing micronutrient metal ions that otherwise would be present in an insoluble or colloidal form. Excessive levels of Cu are toxic to fish[17] and a study[18] indicated that humic substances may be effective in ameliorating toxicity of hydrophobic Cu complexes in fresh water.

Approximately 4–4.5% out of the total 6% K in the HA was soluble in water (Fig. 5). This incomplete dissociation of K may be attributed to the low acid strength (pKa = 15.70) of water, which in this case served as the conjugate acid of the HA salt (K-humate)[19]. Almost all the K in the salt seemed to have been displaced from the exchange sites (carboxylic and phenol)[19] when the salt was acidified with dilute HCl before washing for the functional groups analysis (Fig. 5). The compost contained 20% HA.

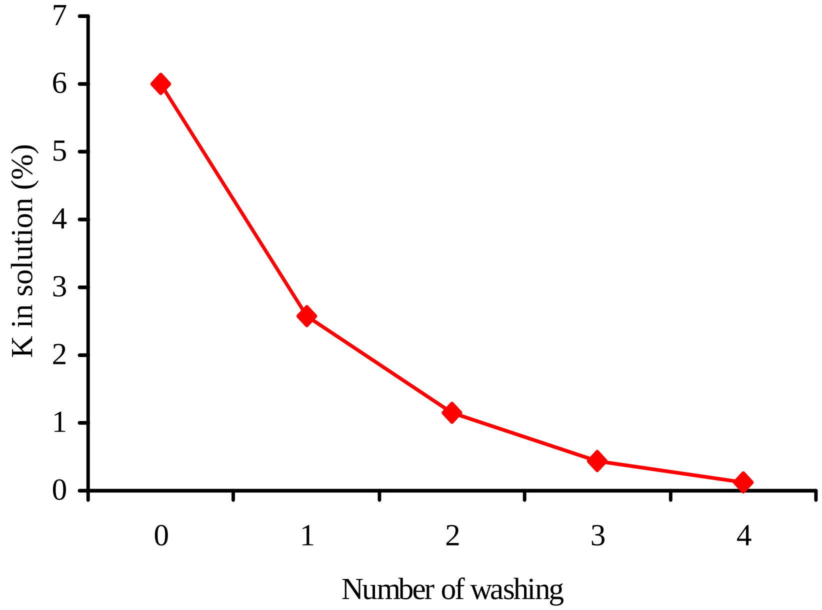

**FIGURE 1**. Effect of washing on K removal from HA.

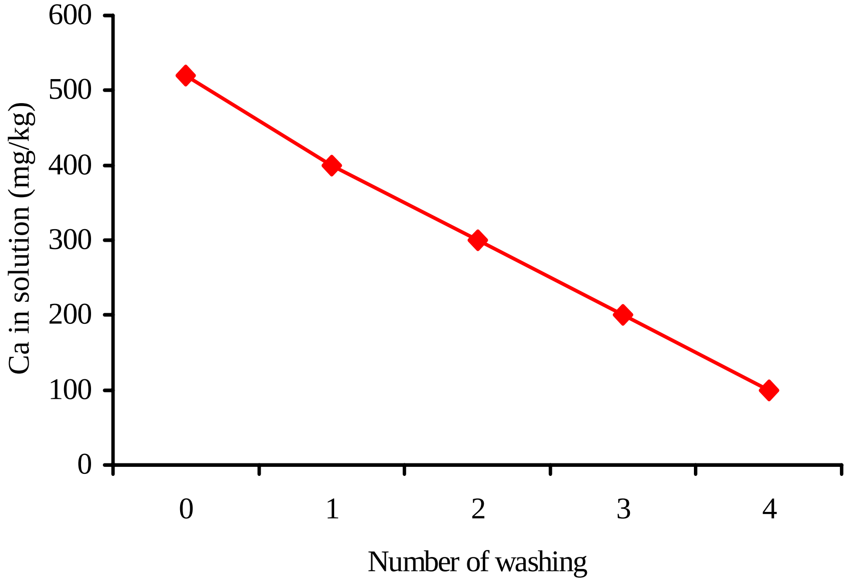

**FIGURE 2**. Effect of washing on Ca removal from HA.

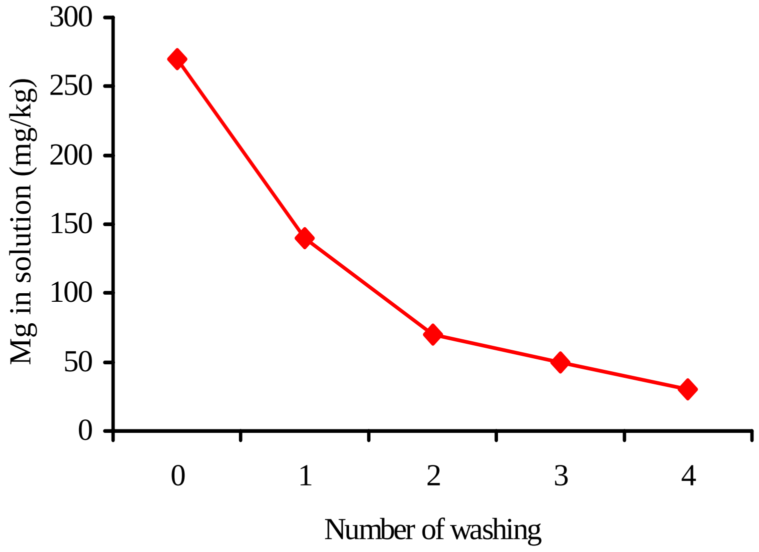

**FIGURE 3**. Effect of washing on Mg removal from HA.

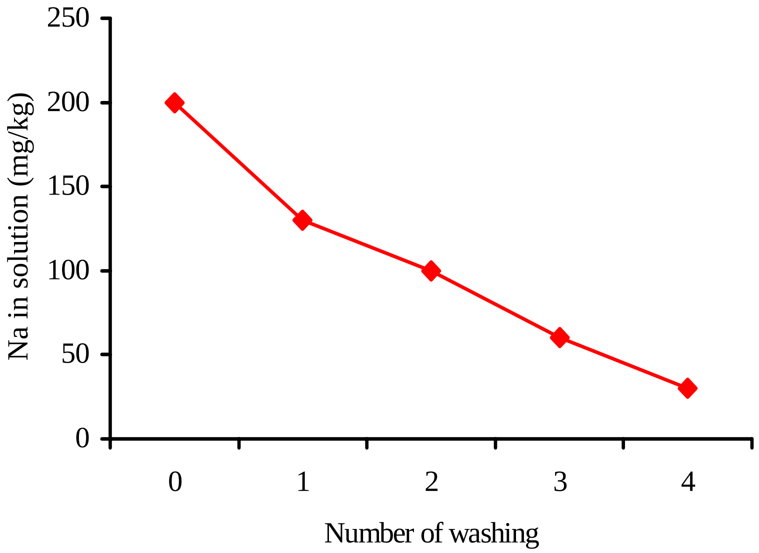

**FIGURE 4**. Effect of washing on Na removal from HA.

It is interesting to note that K in the fulvic acid was relatively high (43%) (Table 1). Perhaps besides serving as K-based fluid fertilizer (K-rich fulvate), this fulvate (pH 1-2) could be used to enhance phosphate rock dissolution particularly in the semi-arid and arid regions where the soil pH is high. Low pH is known to facilitate the dissolution of phosphate rock[20]. By acidulating this material with K-rich fulvate, perhaps potassium phosphate could be produced, an aspect worth investigating but beyond the scope of this study.

The ash content and functional groups of the HA are presented in Table 2. These constituents generally compared well with those reported in the literature[7].

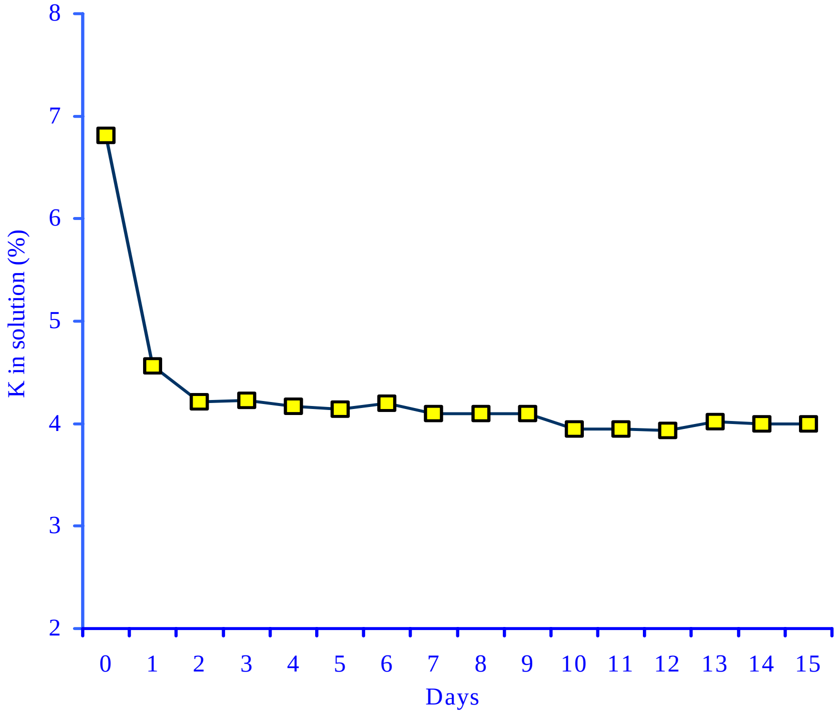

**FIGURE 5**. Equilibrium study of K in HA.

**TABLE 1**
**Some Elemental Concentrations of Fulvic Acid from Composted Pineapple Leaves**

| Element | Pineapple Leaves |
|---|---|
| K | 43% |
| Ca | 620 mg $kg^{-1}$ |
| Mg | 1000 mg $kg^{-1}$ |
| Na | 1200 mg $kg^{-1}$ |

**TABLE 2**
**Some Functional Groups of Humic Acid Derived from Composted Pineapple Leaves**

| Constituent | Information Generated | Literature[7] |
|---|---|---|
| Ash | 0.1% | NA |
| Functional groups | | |
| Total acidity | 673 cmol $kg^{-1}$ | 620–750 |
| COOH | 433 cmol $kg^{-1}$ | 380–450 |
| Phenolic OH | 240 cmol $kg^{-1}$ | 220–300 |

As a means of increasing the K content in HA, the purified HA was reconstituted with K using HA to 0.10 *M* KOH (derived from incinerated pineapple leaves) ratio (weight/volume basis) of 1:25, and this ratio enabled the production of a K-humate containing 38% K and with a pH of 11. Considering the amount of K in the K-humate, the pH, coupled with the fact that the K-humate readily dissolves in water, the possibility of using liquid K-humate as a source of K for fertigation or hydroponics is worth exploring.

## CONCLUSION

Pineapple leaves can be managed by producing compost from these leaves with some high amounts of humic and fulvic acids, which when constituted with K produced from pineapple leaves, can produce K-rich HA and K-rich fulvic acid that can be useful to agriculture, but detailed studies in their use as a source of K for freshwater fish as well as organic fertilizer are required.
